# Draft Genome Sequence of Entomopathogenic *Brevibacillus laterosporus* Strain Lak 1210, an Alkaliphilic Chitin Degrader

**DOI:** 10.1128/genomeA.01251-17

**Published:** 2017-11-16

**Authors:** Lakshmi Prasanna, Tushar Ranjan Moharana, Nizamuddin Sheikh, Veerabhadra Rao Arravapalli, Thangaraj Kumaraswamy, Vincent G. H. Eijsink, Madhusudhana Rao Nalam

**Affiliations:** a Centre for Cellular and Molecular Biology, Hyderabad, India; b Norwegian University of Life Sciences (NMBU), Faculty of Chemistry, Biotechnology and Food Science, Ås, Norway

## Abstract

We announce here the draft genome sequence of *Brevibacillus laterosporus* strain Lak 1210, isolated from mangrove soil. This alkaliphilic strain is an efficient chitin degrader and has the ability to control insects and inhibit phytopathogenic fungi. The assembly consists of 5,082,926 bp, with 4,321 protein-coding sequences and a GC content of 41.15%.

## GENOME ANNOUNCEMENT

The discovery of novel insecticidal genes from non-*Bacillus thuringiensis* strains with improved activity against key pests has led to interest in the entomopathogenic bacterium *Brevibacillus laterosporus* (1). *B. laterosporus* Lak 1210, an alkaliphilic chitinolytic strain with antifungal and insecticidal activity, was isolated from mangrove forest sediments in India (2). Its interesting origin, functional properties, biotechnological potential, and, more importantly, alkaliphilic nature warranted the determination of the genome sequence of strain Lak 1210.

Genome sequencing was performed using the MiSeq platform (Illumina, San Diego, CA, USA) with 2 × 250-bp paired-end reads. The quality of the Illumina raw reads was checked by FastQC (https://www.bioinformatics.babraham.ac.uk/projects/fastqc), and the sequences were processed to remove low-quality bases using Trimmomatic version 0.36 (3). The initial *de novo* genome assembly was performed by coassembly of reads using four different assemblers, Velvet version 1.2.08, MaSuRCA version 2.0.3.1, ABySS version 2.0.2, and SPAdes version 3.5.0 (4–7), and also by an integrated assembly pipeline, A5-miseq version 20150522 (8), which resulted in genome lengths of 5,035,122 bp, 5,064,943 bp, 5,078,237 bp, 5,01,9005 bp, and 5,082,926 bp, respectively. The output of these assemblers was evaluated by QUAST version 4.5 (9). Analysis with QUAST confirmed that A5-miseq gave an optimal assembly of 104 contigs/scaffolds with an *N*_50_ of 672,113 bp. The scaffolding was improved with a final polishing step using SSPACE version 3.0 (10). The first-pass annotation using the Rapid Annotations using Subsystems Technology (RAST) server utilizing GeneMark, Glimmer, and tRNAscan-SE revealed that the genome of strain Lak 1210 is 5,082,926 bp long with a GC content of 41.15% (11).

Functional annotation of the genome by automated annotation using the NCBI Prokaryotic Genome Automatic Annotation Pipeline (PGAAP) predicted a total of 4,623 genes and identified 4,321 coding sequences, 110 tRNAs, and 55 rRNAs (11 5S, 22 16S, and 22 23S), and 133 pseudogenes (12). The final annotation was checked by comparison to the genome sequence of *B. laterosporus* LMG 15441 (NCBI reference sequence NZ_CP007806) using the Mauve Contig Mover version 2.3.1 (http://asap.genetics.wisc.edu/software/mauve). The *B. laterosporus* Lak 1210 genome exhibits 98% identity to the type strain *B. laterosporus* LMG 15441.

Genomic features related to chitinolytic activity included two chitinase genes, two chitin-binding proteins, and a beta-*N*-acetylglucosaminidase. Additional enzymes of biotechnological significance included beta-galactosidase, beta-glucosidase, collagenase, lipases, and proteases. Genome analysis also revealed mosquitocidal toxins, antibiotic resistance genes, and virulence factors. Since chitin degradation by entomopathogens has been investigated only scarcely, current work focuses on the chitinolytic machinery and on biotechnological applications of *B. laterosporus* Lak 1210.

The *B. laterosporus* Lak 1210 genome enriches the genome database of *B. laterosporus* and underpins that strain Lak 1210 has an ability to degrade chitin that might offer value for pest management, biorefining, and disposal of fishery wastes.

### Accession number(s).

The complete genome sequence of *B. laterosporus* Lak 1210 has been deposited in DDBJ/EMBL/GenBank under accession no. NDIP00000000. The version described in this paper is the first version, NDIP01000000. Sequence read data of assembled contigs have been deposited in the NCBI Sequence Read Archive under BioProject no. PRJNA383103.
